# A novel Q-learning algorithm based on improved whale optimization algorithm for path planning

**DOI:** 10.1371/journal.pone.0279438

**Published:** 2022-12-27

**Authors:** Ying Li, Hanyu Wang, Jiahao Fan, Yanyu Geng

**Affiliations:** 1 College of Computer Science and Technology, Jilin University, Changchun, People’s Republic of China; 2 Key Laboratory of Symbolic Computation and Knowledge Engineering of Ministry of Education, Jilin University, Changchun, People’s Republic of China; 3 College of Computer Science, Sichuan University, Chengdu, People’s Republic of China; Torrens University Australia, AUSTRALIA

## Abstract

Q-learning is a classical reinforcement learning algorithm and one of the most important methods of mobile robot path planning without a prior environmental model. Nevertheless, Q-learning is too simple when initializing Q-table and wastes too much time in the exploration process, causing a slow convergence speed. This paper proposes a new Q-learning algorithm called the Paired Whale Optimization Q-learning Algorithm (PWOQLA) which includes four improvements. Firstly, to accelerate the convergence speed of Q-learning, a whale optimization algorithm is used to initialize the values of a Q-table. Before the exploration process, a Q-table which contains previous experience is learned to improve algorithm efficiency. Secondly, to improve the local exploitation capability of the whale optimization algorithm, a paired whale optimization algorithm is proposed in combination with a pairing strategy to speed up the search for prey. Thirdly, to improve the exploration efficiency of Q-learning and reduce the number of useless explorations, a new selective exploration strategy is introduced which considers the relationship between current position and target position. Fourthly, in order to balance the exploration and exploitation capabilities of Q-learning so that it focuses on exploration in the early stage and on exploitation in the later stage, a nonlinear function is designed which changes the value of *ε* in *ε*-greedy Q-learning dynamically based on the number of iterations. Comparing the performance of PWOQLA with other path planning algorithms, experimental results demonstrate that PWOQLA achieves a higher level of accuracy and a faster convergence speed than existing counterparts in mobile robot path planning. The code will be released at https://github.com/wanghanyu0526/improveQL.git.

## Introduction

With technological developments and increasing demand, the scope for mobile robots is becoming more extensive, including applications in machine automation and in fields such as construction, the military, and agriculture [1]. The path planning ability of a mobile robot helps it to navigate, avoid obstacles and find targets [2], and is one of its most important capabilities. The purpose of path planning is to allow the agent to find a safe and collision-free path from the start of a designated area to the end of a designated area [3, 4]. The superiority of path planning technology reflects the intelligence level of a mobile robot. Efficient path planning algorithms can also reduce the loss of mobile robots, which is a prerequisite for intelligent navigation control in unmanned aviation and other systems [5].

Research into robotic motion and path planning began in the mid-1960s [6]. According to the nature of obstacles and targets, path planning is divided into static path planning and dynamic path planning [7]. The positions of obstacles and targets do not change with time during static path planning, but obstacles and targets move freely in the planning area during dynamic path planning.

Path planning is also divided into local path planning and global path planning. The difference is the degree of mastery of environmental information [8]. Local path planning belongs to dynamic path planning and only requires local environmental information to be collected by sensors in real-time to determine the content of the map and the location of nearby obstacles. The methods include artificial potential fields [9], simulated annealing [10], fuzzy logic [11] and neural networks [12], as well as other methods [8]. These methods have their own advantages under certain circumstances, but also face problems such as local minima or local oscillation, slow algorithm speed, large computer storage, and difficulties in determining rules and samples. Therefore, global path planning has become the focus of research as a way of solving these problems. Global path planning belongs to static path planning and needs to master all map information. The methods of global path planning include topological methods, visual graph methods [13], Voronoi diagrams [14], grid methods [15], Dijkstra algorithm, and A* algorithm [16]. Because these methods need exact environmental models, their efficiency is greatly reduced with increases in the complexity and uncertainty of the environment.

Q-learning is another kind of static path planning algorithm [17, 18]. Compared with other static path planning algorithms, Q-learning has a strong learning ability and adapts to complex unknown environments. Therefore, a large number of researchers have studied Q-learning in path planning.

Q-learning is a kind of reinforcement learning algorithm. Path planning based on reinforcement learning, using algorithms similar to Q-learning, has become a hotspot in current research due to the increasing demand for intelligent applications of mobile robots [19]. Reinforcement learning uses a method similar to trial and error in human thinking to find an optimal behavior strategy [20]. Path planning based on reinforcement learning maps the environmental state perceived by sensors to the actions of actuators and responds quickly to changes in the external environment in order to achieve autonomous path planning with the advantages of real-time reactions and speed. Path planning algorithms based on reinforcement learning include Temporal Difference [21], the Q-learning algorithm, Sarsa algorithm [22], Dyna algorithm [23], and Actor-Critic [24]. Temporal Difference is an earlier reinforcement learning algorithm and the basis for improvements to the Q-learning and Sarsa algorithms. The difference between Q-learning and Sarsa is whether iteration is based on the state-value function or the action-value function. Compared with other algorithms, Q-learning reinforcement learning algorithms have a strong learning ability and adapt to complex unknown environments. The *ε*-greedy Q-learning method is the most common and widely used.

However, Q-learning also has limitations. As the complexity of the environment increases, the Q-value matrix grows exponentially. The update of the Q-value matrix requires more storage space and a longer calculation time. In order to solve the navigation problem of mobile robots and reduce the amount of memory required by the algorithm, researchers have proposed improvements based on Q-tables or alternative strategies. As regards alternative strategies, Shi et al. [25] used Q-learning for adaptive servo gains adjustment, and proposed a fuzzy-based method for tuning the learning rate in order to improve Q-learning performance. Li et al. [26] proposed a novel off-strategy interleaved Q-learning algorithm by introducing behavior control strategy. As regards Q-table improvements, Wang and de Silva [27] proposed a new distributed Q-learning algorithm, which updates a Q-table with local rewards to reduce Q-learning spaces. Song et al. [28] applied a dynamic wave expansion neural network to specified initializations of Q-values in a Q-table, which improved the convergence efficiency of Q-learning. Konar et al. [29] reduced the number of repeated updates of a Q-table by assuming the distance from the current state to the next state and the target, thereby reducing the time complexity of the algorithm.

However, Q-learning with the above improvements still needs to calculate all possible action-states, and therefore it still has the disadvantage of slow convergence speed. To solve this problem, metaheuristic optimization algorithms have been applied to improve the initialization phase of Q-learning. This provides a better initial state for Q-learning, and reduces the amount of time required for calculation and subsequent convergence.

A number of metaheuristic optimization algorithms have been proposed. Kennedy and Eberhart [30] proposed Particle Swarm Optimization, which originates from the predatory behavior of birds and seeks an optimal solution through collaboration and information sharing between individuals in the population. Passino [31] proposed Bacterial Foraging Optimization, which is a bionic random search algorithm that imitates the behavior of E. coli swallowing food in the human intestine. Rashedi et al. [32] proposed the Gravitational Search Algorithm, which uses the gravitational force between particles in the population to guide the movement of each particle to find the optimal solution. Yang [33] proposed the Bat Algorithm, which is a heuristic algorithm simulating bats in nature. The Bat Algorithm is mainly focused on searching for prey and avoids obstacles by simulating ultrasound to find the global optimal solution. Mirjalili [34] proposed Moth-Flame Optimization, a swarm intelligence optimization algorithm inspired by natural laws to simulate the spiral flight path of moths based on their navigational mechanism during flight. Mirjalili et al. [35] proposed the Gray Wolf Optimizer, which is an optimized search method based on the social hierarchy of gray wolves and inspired by their predatory activities. Inspired by the precise navigation of birds over long-distance aerial paths, Zamani et al. [36] proposed a novel differential evolution algorithm named the Quantum-based Avian Navigation Optimizer Algorithm (QANA). Mohammad et al. [37] proposed an efficient binary version of the QANA named BQANA to solve the feature selection problem of high-dimensional medical datasets. To solve engineering optimization challenges, Zamani et al. [38] proposed the Starling Murmuration Optimizer, which is based on the behavior of starlings during their stunning murmurations. Mirjalili and Lewis [39] proposed the Whale Optimization Algorithm (WOA) to simulate the spiral hunting behavior of humpback whales.

WOA has the advantages of a relatively simple concept, does not require gradient information, and is easy to implement. Therefore, in this paper WOA is selected to optimize the initial Q-table. However, similar to other population-based heuristic algorithms, WOA still has the problem of slow convergence speed and low convergence accuracy. Mafarjaa et al. [40] combined WOA with Simulated Annealing (SA), and implemented feature selection by embedding SA into WOA. Mafarjaa et al. [41] used WOA with an improved stochastic process or WOA with crossover and mutation operators in feature selection. Kaveh and Ghazaan [42] improved the original formula of WOA to enhance the convergence speed and increase the level of accuracy of the original algorithm. Combining WOA with a local search strategy, Abdel-Basset et al. [43] proposed the Hybrid Whale Algorithm to solve the problem of shop scheduling. To balance the capabilities of exploration and exploitation in WOA, Kaur and Arora [44] introduced chaos theory into WOA and proposed the Chaotic Whale Optimization Algorithm to improve and enhance the performance of the original WOA. Mohammad et al. [45] proposed an Enhanced Whale Optimization Algorithm (E-WOA) using a pooling mechanism and three improved search strategies named migrating, preferential selecting, and enriched encircling prey. E-WOA was applied to medical datasets to verify the effectiveness of the algorithm, especially to detect coronavirus disease (COVID-19) in 2019. Mohammad et al. [46] proposed an efficient hybrid algorithm that combined WOA with an improved Month-Flame Optimization algorithm to solve the optimal power flow problem in power systems.

However, path planning methods for mobile robots using *ε*-greedy Q-learning still have three defects. First, Q-learning initializes a Q-table to zero at the time of initialization, increasing the time to calculate and update the Q-table, and subsequently resulting in a slow convergence process. Second, the strategy of *ε*-greedy Q-learning selects the next state in the exploration process too randomly, which means too much time is wasted in the exploration process. Third, because the value of *ε* is fixed, *ε*-greedy Q-learning path planning cannot switch processes flexibly between exploration and exploitation under any circumstances.

In order to solve the above problems, this paper proposes a new path planning algorithm named the Paired Whale Optimization Q-learning Algorithm (PWOQLA), which is based on an improved WOA and an improved *ε*-greedy Q-learning. Firstly, in order to correct the shortcoming of slow convergence caused by Q-learning initialization, WOA, as a metaheuristic optimization algorithm, is chosen for Q-table initialization instead of simply setting the values of a Q-table to zero. In this way, a Q-table that contains previous experience is learned before the exploration process. Thus, in the subsequent Q-learning path planning, the calculation time is reduced and a path with fewer steps and smoother corners is obtained.

Secondly, based on the pairing behavior of whales, a Paired Whale Optimization Algorithm (PWOA) is proposed to accelerate the convergence speed of WOA. The main innovation of PWOA is to pair each whale when initializing the population. When one paired whale finds a prey position, the position of the other paired whale is updated to the same prey position. The result of this improvement is to accelerate the speed of the whale population approaching a local optimal solution. Compared with the original WOA, PWOA further improves the convergence speed of Q-learning when initializing the Q-table.

Thirdly, to improve the convergence efficiency of *ε*-greedy Q-learning, which uses a random exploration strategy, a novel selective exploration strategy (SES) is proposed based on the relationship between current agent position and target position. During each exploration, the agent judges the relationship between those two positions. Based on the judgment of the relationship, the agent will selectively explore two directions that are closer to the target position, instead of exploring four directions at random. SES reduces the number of useless explorations to achieve the purpose of accelerating the convergence speed of *ε*-greedy Q-learning.

Fourthly, in order to switch flexibly between exploration process and exploitation process, we propose a nonlinear function that changes the value of *ε* in *ε*-greedy Q-learning dynamically based on the number of iterations. In other words, the exploitation probability of *ε*-greedy Q-learning gradually increases as the number of iterations increases, whereas the exploration probability of the surrounding environment decreases. Therefore, by changing the value of *ε* dynamically, exploration and exploitation can be switched flexibly.

Finally, combining the above improvements to Q-learning and WOA, the result is the proposed PWOQLA. In PWOQLA, PWOA is applied to the Q-table initialization phase of the improved Q-learning. Compared with the original Q-learning algorithm, PWOQLA is more accurate and more efficient at robot path planning, and experimental results show that the proposed algorithm has a greater level of accuracy and faster convergence compared to several path planning algorithms with similar functions.

The rest of this paper is structured as follows. The second section introduces *ε*-greedy Q-learning and WOA. The third section introduces the working principles and steps of PWOQLA. The fourth section compares PWOQLA with similar algorithms and discusses the experimental results. The fifth section draws the conclusions.

## Related work

### Q-learning

Q-learning [17] is a model-less algorithm that is one of the main reinforcement learning algorithms. In the Markov environment, Q-learning has the ability to learn and provides an intelligent system to select the best action using experienced action sequences. Q-learning learns through the Q-value function. In the Q-value function, the state transition probability and the next status decide the current state and the selected action, and the agent receives an instant return after the selected action. The strategy of Q-learning is to find maximum rewards way into the future. Q-learning is called model-less because it compares the expected values of actions without an environmental model, and this is the advantage of Q-learning.

In Q-learning, each Q (*s*, *a*) has a corresponding Q-value. In the subsequent learning process, the next action is selected according to Q (*s*, *a*). The sum of the rewards obtained from executing a certain strategy and performing the current action is defined as the Q-value. The optimal Q-value is defined as the sum of the rewards acquired by executing related actions and executed according to the optimal strategy, which is defined as follows:

$$Q(s_{t},a_{t})=(1-\alpha )Q(s,a)+\alpha [r_{t+1}+\gamma \underset{a_{t+1}}{\max}Q(s_{t+1},a_{t+1})]$$ (1)

In Eq (1), *s*_*t*_ is the current state; *a*_*t*_ is the action performed in state *s*_*t*_; *r*_*t*+1_ is the reinforcement signal received after *s*_*t*_ is executed and is also called the reward; *s*_*t*+1_ is the next state; *γ* is a discount factor (0 ≤ *γ* < 1); and *α* is a learning coefficient (0 < *α* < 1).

Each agent learning process can be considered as starting from a random state and adopting an *ε*-greedy strategy or Boltzmann distribution strategy to select the next actions. The *ε*-greedy strategy is used in decision making. For example, when *ε* is initialized to 0.9, it means that there is a 90% probability that the agent will choose a behavior according to the optimal value of the Q-table, and a 10% probability of choosing a random selection. To allow the agent to search for all possible actions and update each Q (*s*, *a*) for each action, the random selection strategy is adopted. The agent observes the new state after executing the selected action. The Q (*s*, *a*) of the previous state and action is then updated in response to the maximum Q-value and the return of the new state. Based on the new state, the agent will continue to choose actions until it reaches the end state.

### Whale Optimization Algorithm

The Whale Optimization Algorithm (WOA) is a heuristic optimization algorithm proposed by Mirjalili Seyedali [39]. The algorithm proceeds as follows. In the search space for the optimization problem, each humpback whale is a candidate solution, called a search whale. A set of search agent whales is used to find the global optimum of an optimization problem in WOA. For a given problem, the search process starts from a random solution when initializing and then updates candidate solutions according to optimization rules until the final criterion is met. In fact, WOA simulates the behavior of humpback whales looking for and attacking prey.

#### Encircling prey

The humpback whale recognizes the location of its prey and surrounds the prey. Because the prey position of the optimal solution is a priori unknown, the target prey position is presumed to be the current optimal solution, and other search whales update their positions through the “target prey”. The mathematical model of prey behavior is as follows:

$$\vec{D}=|\vec{C}\cdot \vec{G}(t)-\vec{X}(t)|$$ (2)

$$\vec{X}(t+1)=\vec{G}(t)-\vec{A}\cdot \vec{D}$$ (3)

In the above, $\vec{X}(t)$ is the current position vector; $\vec{G}(t)$ is the current optimal solution position vector; $\vec{D}$ is the distance between the search whale and the target prey; *t* is the current number of iterations; and $\vec{A}$ and $\vec{C}$ are coefficient vectors.

If there is a better optimal solution position vector, $\vec{G}(t)$ should be updated in the current iteration. The formulae for calculating $\vec{A}$ and $\vec{C}$ are as follows:

$$\vec{A}=2\vec{a}\cdot {\vec{r}}_{a}-\vec{a}$$ (4)

$$\vec{C}=2\cdot {\vec{r}}_{c}$$ (5)

In the above, ${\vec{r}}_{a}$, and ${\vec{r}}_{c}$ are random vectors in the range [0, 1], and $\vec{a}$ decreases linearly from 2 to 0 during the iteration.

### Bubble-net strategy

Humpback whales move around their prey in a spiral path and simultaneously spit out bubbles to create traps. This is known as the bubble-net strategy for hunting prey. In the WOA model, the contraction and surround mechanism is achieved by reducing $\vec{a}$ in Eq (4). The fluctuation range of $\vec{A}$ decreases with decreases in $\vec{a}$. According to Eq (4), $\vec{A}$ is a random value in the interval [−a, a]. Setting $\vec{A}$ to be a random value in the interval [−1, 1], the position of the search whale will move randomly to any position between the current optimal solution position and the previous position. The new position of the search whale is calculated as follows:

X→(t+1)=D→'⋅ebl⋅cos(2πl)+G→(t) (6)

D→'=|G→(t)−X→(t)| (7)

In the above, D→' means the best solution obtained so far, which represents the distance from the whale to the prey; *l* is a random number in the range [−1, 1]; and *b* is the constant of the logarithmic spiral shape and can be set to different values according to specific application scenarios.

The humpback whale swims along a spiral path toward its prey. To update the whale’s predatory position, the mathematical model of the whale’s spiral path is as follows:

X→(t+1)={G→(t)−A→⋅D→,p<0.5G→(t)+D→'⋅ebl⋅cos(2πl),p≥0.5 (8)

In Eq (8), the variable *p* is a random number between 0 and 1.

#### Searching for prey

As well as using the bubble-net strategy in the exploitation process, humpback whales also need to search for prey randomly in the exploration process. The mathematical model of searching for prey is as follows:

$$\vec{D}=|\vec{C}\cdot \vec{X_{rand}}(t)-\vec{X}(t)|$$ (9)

$$\vec{X}(t+1)=\vec{X_{rand}}(t)-\vec{A}\cdot \vec{D}$$ (10)

In the above, $\vec{X_{rand}}(t)$ is the position vector of a search whale randomly selected from the population.

In order to ensure exploration and convergence, when $|\vec{A}|\geq 1$, the randomly selected search whale becomes the key point when other whales update the position. In other cases ($|\vec{A}|<1$), the current optimal solution position plays a pivotal role in updating other search whales.

## Methods

The Paired-Whale Optimization Q-learning Algorithm (PWOQLA) is a path planning algorithm that uses an improved WOA to initialize the Q-value of an improved *ε*-greedy Q-learning. The aim of PWOQLA is to overcome the disadvantage of slow convergence in the original *ε*-greedy Q-learning. The first part of this section introduces the Whale Optimization Q-learning Algorithm (WOQLA), which combines the algorithms of the original Q-learning and original WOA. The second part introduces the process of improving WOA, and the third part introduces the process of improving *ε*-greedy Q-learning. The final part introduces the application of PWOQLA in path planning.

### Whale Optimization Q-learning Algorithm

The Whale Optimization Q-learning Algorithm (WOQLA) is an algorithm for mobile robot path planning that combines the original WOA with Q-learning initialization. To overcome the shortcomings of Q-learning, such as slow convergence caused by initialization, WOQLA optimizes the initialization of the Q-table instead of simply setting the Q-values to zero.

In the initialization phase, WOQLA generates a number (n) of whale populations in a 20 × 20 grid space and uses the Q-value calculation in Eq (1) to calculate the fitness value of each whale. The position with the highest Q-value represents the best whale position. The WOA is then used to optimize the Q-value of each whale in the whale population. When the maximum number of iterations is reached, the initialization of the Q-table by WOA ends. The original ε-greedy Q-learning is then used for path planning according to the newly obtained Q-table. Based on the newly initialized Q-table, the Q-value calculation formula is used in the iterative update of the Q-table. After the iteration is completed, the final Q-table is obtained. From this Q-table, we can find the path with the largest Q-value, which represents the best path. In this way, the Q-table containing previous experience is learned through WOA before the Q-learning algorithm searches, which helps to reduce the subsequent calculation time and accelerates the speed of Q-learning convergence.

### Paired-Whale Optimization Algorithm

**Fig 1:**
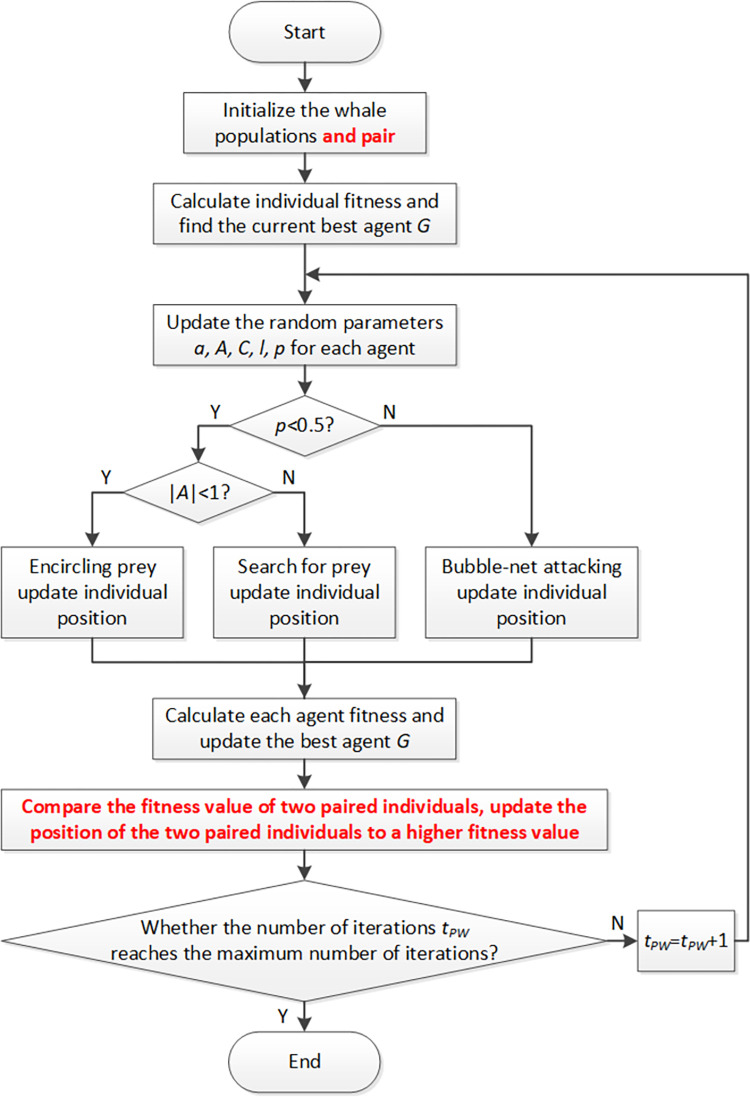
The flow chart of PWOA.

WOA has the problems of slow convergence and low levels of accuracy. Inspired by research on humpback whales and the observation that they perform activities mostly in pairs [47–50], this paper proposes the Paired-Whale Optimization Algorithm (PWOA) based on the pairing behavior of humpback whales. Pairing behavior helps paired whale individuals to find food faster with the help of their peers, which accelerates the convergence speed of the original WOA. The main improvement of PWOA compared with the original is that the algorithm finds a mate for each whale when initializing the whale population, so that each whale has another whale paired with it. The pairing strategy is pairwise pairing in the order of randomly generated whale individuals. The original WOA is then executed. At each iteration, the fitness value of each whale is calculated. When whale *G* is found to be in the best position, the algorithm compares the fitness value of whale *G* with the fitness value of the pair of whale *G*. If the fitness value of whale *G* is large, the position of the paired whale is updated to the position of whale *G*. If the fitness value of whale *G* is small, the position of whale *G* is updated to the position of the paired whale. In this way, in each cycle, each pair of whales chooses the better position of the two to find their prey at the same time, then conducts their own exploration or exploitation process respectively. The current number of iterations in PWOA is *t*_*PW*_. The result is that the convergence speed of the final algorithm is accelerated. Such improvements accelerate the speed of the whale population approaching the local optimal solution, and also accelerate the convergence of the final algorithm. The pseudo-code of the PWOA algorithm is shown in Algorithm 1, and a flowchart is shown in Fig 1.

**Algorithm 1** Pseudo-code of PWOA

Initialize the whales population *Xi(i = 1*,*2*,…,*n)*

Pair whales in the population

Calculate the fitness of each search agent

*G* = the best search agent

**while** (*t* < maximum number of iterations)

    **for** each search agent

        Update *a*, *A*, *C*, *l*, and *p*

        **if** (*p*<0.5)

            **if** (|*A*| < 1)

                Update the position of the current search agent by the Eq (2)

            **else if** (|*A*|≥1)

                Select a random search agent (*X*_*rand*_)

                Update the position of the current search agent by the Eq (10)

            **end if**

        **else if** (*p*≥0.5)

            Update the position of the current search by the Eq (6)

        **end if**

    **end for**

    Check if any search agent goes beyond the search space and amend it

    Calculate the fitness of each search agent

    Update *G* if there is a better solution

    **for** each paired individuals

        Compare the fitness value of two paired individuals

        Update the position of the two paired individuals to a higher fitness value

    **end for**

    *t = t+1*

**end while**

return *G*

### Improved Q-learning

**Fig 2:**
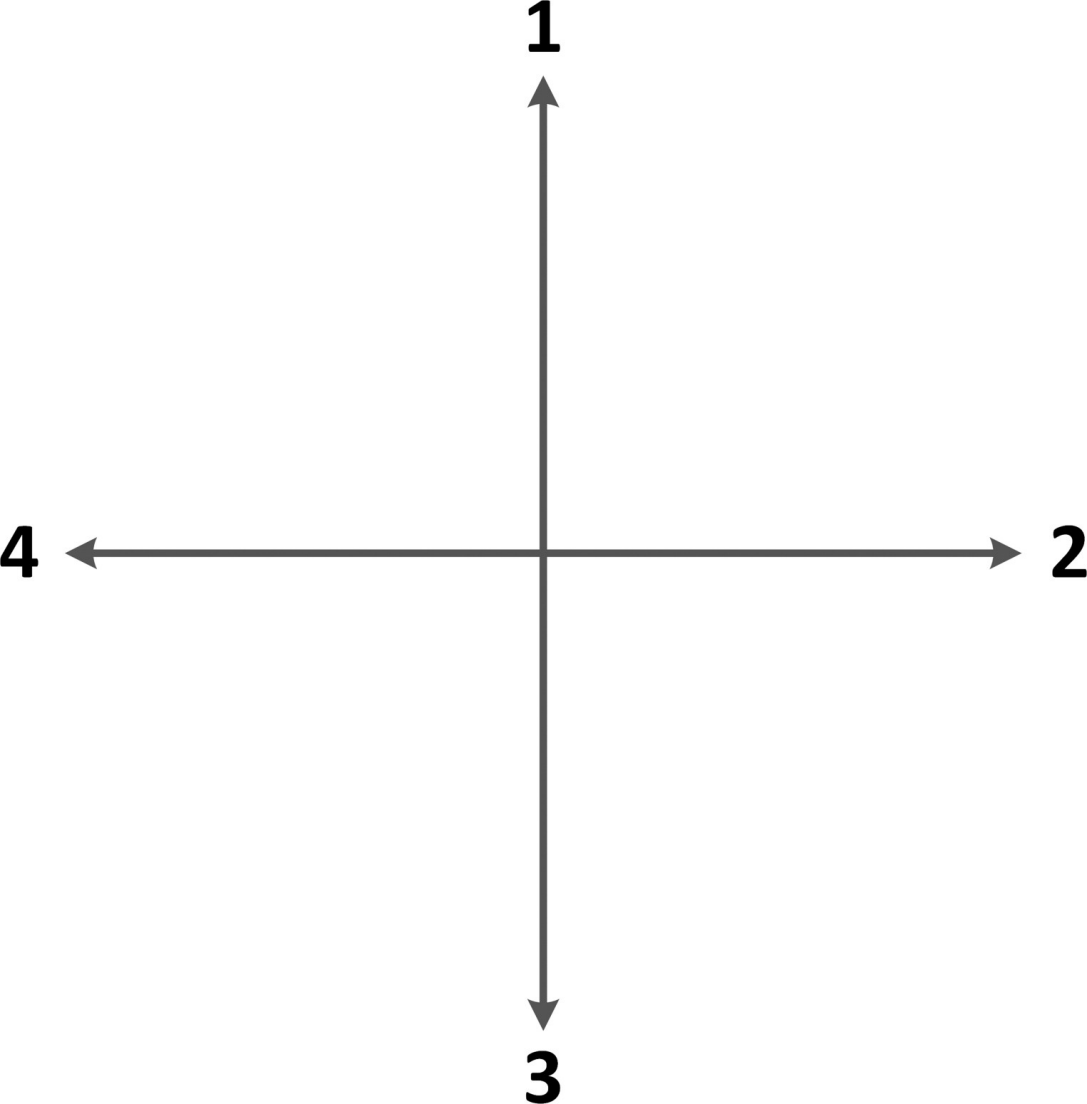
Random action direction.

In order to simplify a real-world application scenario, a 20 × 20 grid space is used to model the real environment. We assume that each grid position corresponds to a corresponding coordinate in real space. The value of each grid is mapped to the Q-table of *ε*-greedy Q-learning. When the reward is −1, the grid is an obstacle. When the reward is +1, the grid is free space. When the reward is 100, the grid is the target position. As shown in Fig 2, an agent has four random actions at position *s*_*t*_: action 1 goes up; action 2 goes right; action 3 goes down; and action 4 goes left.

In order to improve ε-greedy Q-learning, firstly, this paper proposes a novel selective exploration strategy (SES) based on the target position, with the aim of improving the convergence efficiency of the original *ε*-greedy Q-learning and to reduce the number of useless explorations. During each exploration, the agent first judges the relationship between the current agent position *s*_*t*_*(x*_*t*_,*y*_*t*_*)* and the target agent position *s*_*g*_*(x*_*g*_,*y*_*g*_*)*. The agent will then explore two directions closer to the target position based on its judgment of the position relationship, instead of randomly exploring four directions. The exploration rules of the SES are as follows:

If *x*_*t*_≤*x*_*g*_ and *y*_*t*_<*y*_*g*_, then *a* =*rand*(1,2); if *x*_*t*_<*x*_*g*_ and *y*_*t*_≥*y*_*g*_, then *a* = *rand*(2,3).

If *x*_*t*_>*x*_*g*_ and *y*_*t*_≤*y*_*g*_, then *a* = *rand*(3,4); if *x*_*t*_≥*x*_*g*_ and *y*_*t*_>*y*_*g*_, then *a* = *rand*(1,4).

Secondly, in order to switch flexibly between exploration and exploitation process, the ε value in ε-greedy Q-learning is changed dynamically. The equation for calculating the value of *ε* is as follows:

$$\varepsilon =u+v/(1+e^{a-bt})$$ (11)

**Fig 3:**
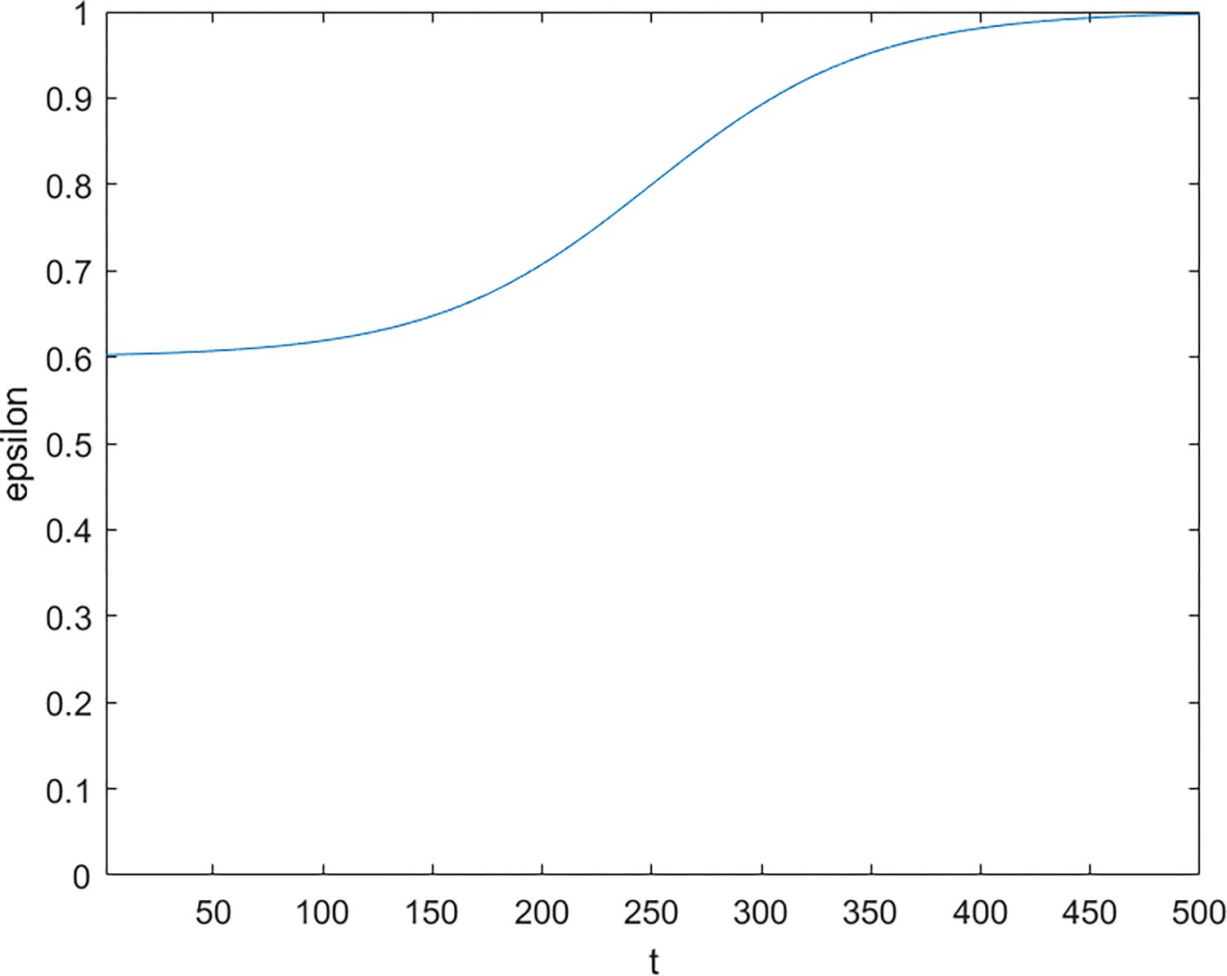
The curve of parameter *ε*.

In Eq (11), *u* is the value of *ε* at the beginning of the iteration, and *v* is the incremental range of *ε*. The sum of *u* and *v* is set to 1, and *u* and *v* are temporarily set to 0.6 and 0.4 respectively. The variables *a* and *b* are the coefficient parameters of the dynamic curve, and their values are determined by the number of iterations. The variable *t* is the current number of iterations. The change curve of *ε* is shown in Fig 3.

It can be deduced from Eq (11) that the changing trend of *ε* is decreasing. Therefore, as the number of iterations increases, the development probability of *ε*-greedy Q-learning is gradually increased, and the probability of exploring the surrounding environment is reduced. Consequently, the early stage attends to exploration capability whereas the later stage attends to exploitation capability.

To summarize these two improvements, firstly, we reduce the number of useless explorations by referring to the relationship between the target and the current position through the SES strategy; and secondly, the proportion of exploration and exploitation in different periods is planned reasonably with the *ε* curve. These two improvements help to reduce the amount of calculation in the algorithm and save computing resources.

### Paired-Whale Optimization Q-learning Algorithm

**Fig 4:**
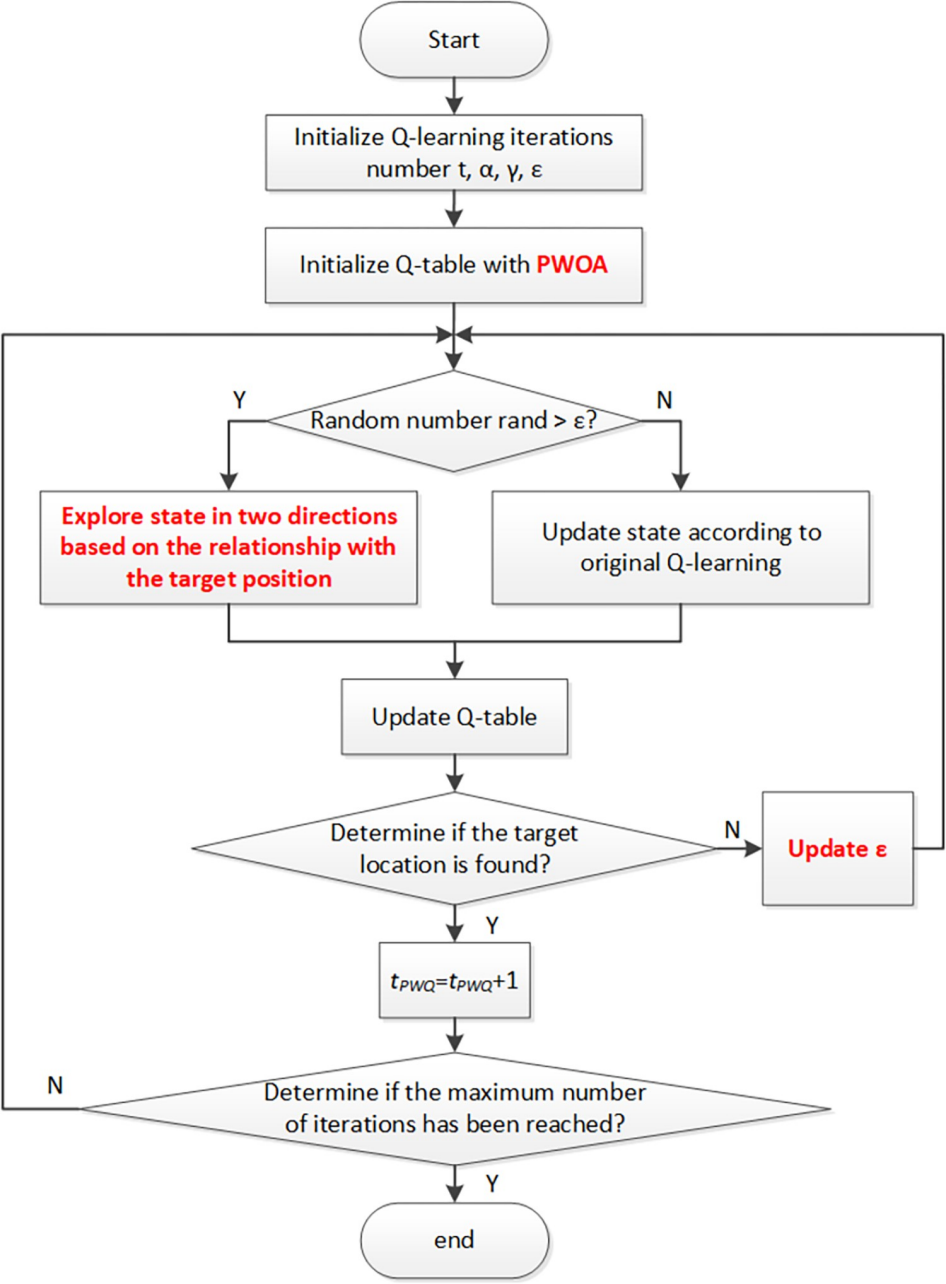
The flow chart of PWOQLA.

The Paired-Whale Optimization Q-learning Algorithm (PWOQLA) is a path planning algorithm that uses PWOA to initialize the Q-table in improved Q-learning, instead of simply initializing the Q-table to zero. During initialization, a number of whale populations (*n*) is generated and matched in a 20 × 20 grid space. The Q-value calculation in Eq (1) is used to calculate the fitness value of each whale. The position with the highest Q-value represents the best whale position. PWOA is then used to optimize the Q-value of the whale population. After the number of iterations reaches the maximum, Q-table initialization is completed. Subsequent path planning is performed using the improved *ε*-greedy Q-learning based on the newly obtained Q-table. The current number of iterations in PWOQLA is *t*_*PWQ*_, and the final result represents the best path. The pseudo-code of PWOQLA is shown in Algorithm 2, and a flowchart of the algorithm is shown in Fig 4.

**Algorithm 2** Pseudo-code of PWOQLA

Initialize Q-values in Q-table with PWOA, start position, target position

Select a starting state *Q(s*_*1*_, *a*_*1*_*)*

**while** (*t* < maximum number of iterations)

    **while** *s*_*t*_ is not target position

        **if** (Probability < *ε*)

            Choose *a*_*t*_ according to the maxQ(s_t_)

            Take action *a*_*t*_, and return reward *r*

            Update Q-value by the Eq (1)

            Move to new state

        **else if** (Probability ≥ *ε*)

            Randomly choose *a*_*t*_ in two directions according to the relationship with the target

        **end if**

        Update *ε* by the Eq (11)

    **end while**

**end while**

## Experiment

In this section, we simulate the effect of PWOQLA in mobile robot path planning in a grid environment with 20 × 20 obstacles. The original Q-learning, Improved Decentralized Q-learning (IDQ) [51], A* algorithm [52], and WOQLA are compared with PWOQLA to verify the effectiveness of PWOQLA in path planning. The A* algorithm is one of the basic algorithms for path planning. IDQ is one of the classical algorithms for improving Q-learning and is often used as a comparison algorithm.

### Experimental environment and parameters

The experiment is performed in a 20 × 20 grid. The number of simulations is set to 30 as this number of simulation samples represents the general sample-generation quantity that is sufficient to measure the performance of the algorithm [30, 33, 39]. The side length of each grid is a standard unit, and there is no fixed definition here. As shown in Fig 2, a mobile robot has four motion directions: action 1 moves forward, action 2 moves right, action 3 moves backward, and action 4 moves left. The reward and punishment rules are the same in Q-learning, IDQ, WOQLA, and PWOQLA. If the mobile robot encounters an obstacle, the penalty Q-value is reduced by 1; if the mobile robot moves to free space, the reward Q-value is increased by 1; and if the mobile robot moves to find the target position, the reward Q-value is increased to 100. Finally, a 400 × 4 Q-table containing all the information on 400 positions and 4 actions at each position is established.

In Q-learning, *α* is the learning coefficient, and reflects the degree to which the previous Q-value is retained. Following the work of Khriji et al. [53], *α* is set to 0.2 in this study. The variable *γ* is the discount factor, which represents how the agent treats future rewards when it receives the current reward. In this study, *γ* is set to 0.8 [53]. The number of iterations *t* in Q-learning is set to 500. In WOA, ${\vec{r}}_{a}$, ${\vec{r}}_{c}$ are random vectors in the range [0, 1], and *a* decreases linearly from 2 to 0 during the iteration. The parameter *b* is a constant defining the logarithmic spiral shape, which is set to 1 in this study [54]; and *l* is a random number in the range [−1, 1]. The variable *p* is a random number between 0 and 1. The population number *n* of WOA is set to 30, and the number of iterations *t*_*PW*_ and *t*_*PWQ*_ are both set to 500. The parameter settings are shown in Table 1.

**Table 1:** The simulation parameters.

Algorithm	Parameter	Value
Q-learning	*α*	0.2
	*γ*	0.8
	*t*	500
WOA/PWOA/PWOQLA	${\vec{r}}_{a}$	random between [0, 1]
	${\vec{r}}_{c}$	random between [0, 1]
	*a*	decreases linearly from 2 to 0
	*b*	1
	*l*	random between [–1,1]
	*p*	random between [0, 1]
	*n*	30
	*t*_*PW*_	500
	*t*_*PWQ*_	500

The experiment was conducted on an Intel (R) Core (TM) i7-7500U CPU @ 2.70 GHz platform. The graphics card was an Intel (R) HD Graphics 620, and the simulation software was MATLAB R2020a.

### Experimental results and analysis of PWOA

By solving 8 classic benchmark functions used in the optimization problem, the efficiency of the PWOA is compared with the original WOA. In this experiment, the population size is 30 for both WOA and PWOA, and the maximum iteration is set to 500. The benchmark functions used in the experiment include three types: unimodal (F1, F2, F3); multimodal (F4, F5, F6); and fixed-dimension multimodal (F7, F8) [55]. Table 2 summarizes the details of the benchmark functions in these experiments, including the cost function, the number of design variables V_no, the range of optimization variables, and the optimal value *f*_min_.

**Table 2:** Description of composite experimental classical benchmark functions.

Function	V_no	Range	*f*_min_
$F_{1}(x)=\sum_{i=1}^{n}x_{i}^{2}$	30	[–100,100]	0
$F_{2}(x)=\sum_{i=1}^{n}\left|x_{i}\right|+\prod_{i=1}^{n}\left|x_{i}\right|$	30	[–10,10]	0
$F_{3}(x)=\sum_{i=1}^{n}ix_{i}^{4}+random[0,1)$	30	[-1.28,1.28]	0
$F_{4}(x)=\sum_{i=1}^{n}\left[x_{i}^{2}-10\cos(2\pi x_{i})+10\right]$	30	[-5.12,5.12]	0
$F_{5}(x)=-20\exp(-0.2\sqrt{\frac{1}{n}\sum_{i=1}^{n}x_{i}^{2}})-\exp(\frac{1}{n}\sum_{i=1}^{n}\cos(2\pi x_{i}))+20+e$	30	[−32,32]	0
$F_{6}(x)=\frac{1}{4000}\sum_{i=1}^{n}x_{i}^{2}-\prod_{i=1}^{n}\cos(\frac{x_{i}}{\sqrt{i}})+1$	30	[–600,600]	0
$F_{7}(x)={\left(x_{2}-\frac{5.1}{4\pi^{2}}x_{1}^{2}+\frac{5}{\pi }x_{1}-6\right)}^{2}+10(1-\frac{1}{8\pi })\cos x_{1}+10$	2	[–5,5]	0.398
$F_{8}(x)=-\sum_{i=1}^{4}c_{i}\exp(-\sum_{j=1}^{3}a_{ij}{\left(x_{j}-p_{ij}\right)}^{2})$	3	[1,3]	−3.86

**Fig 5:**
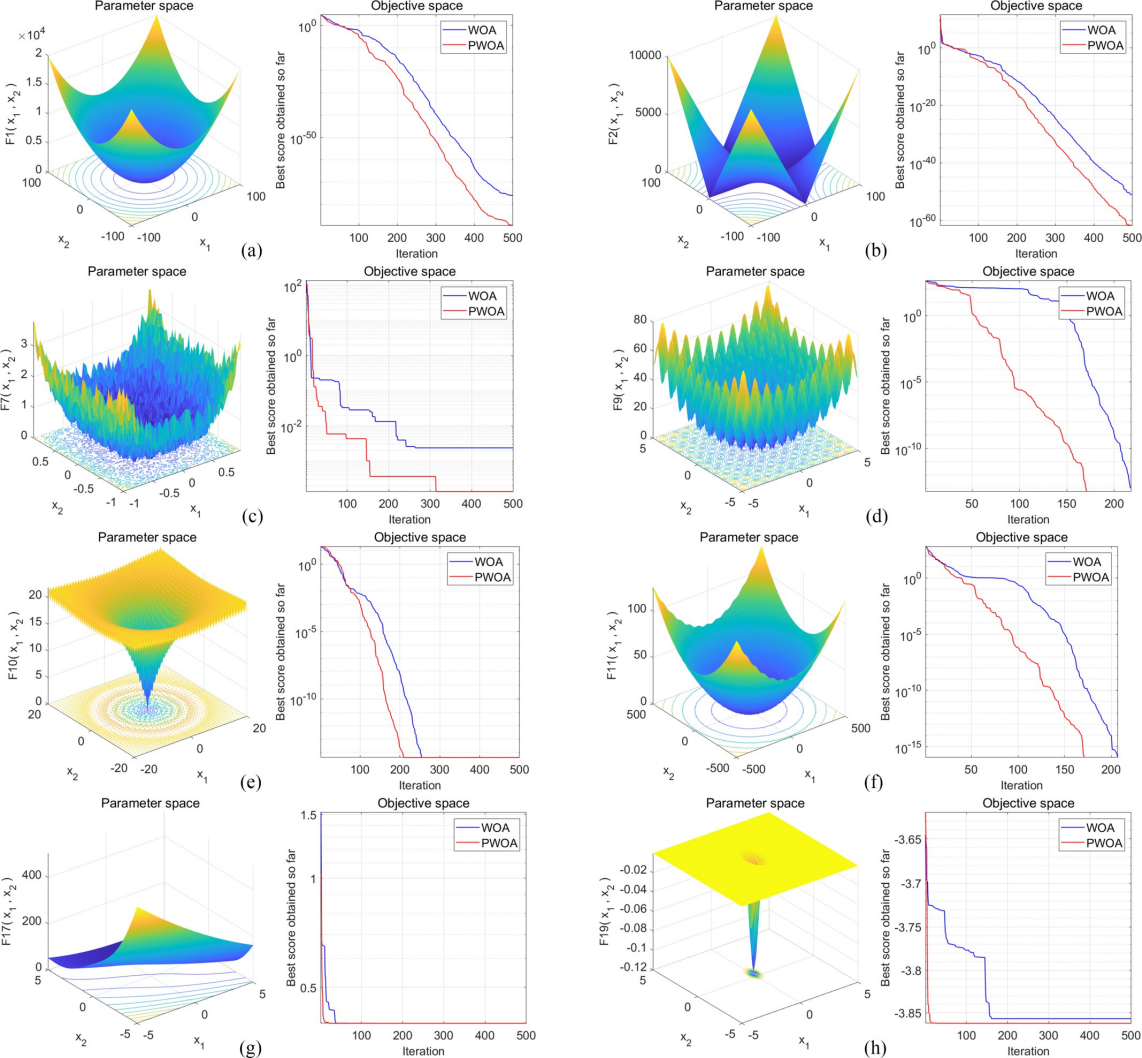
Comparison of convergence curves of WOA and PWOA obtained in experimental classical benchmark functions.

The algorithms WOA and PWOA are run 30 times each for every benchmark function, starting with different populations randomly generated. Fig 5 shows a typical two-dimensional function graph of the test cases selected in the experiments and compares the changes in the convergence curves of WOA and PWOA obtained on the benchmark functions. The statistical results are listed in Table 3, which include the average and standard deviation of the test cases.

**Table 3:** Comparison of optimization results obtained for experimental classical benchmark functions.

F	WOA		PWOA	
	Average	Standard Deviation	Average	Standard Deviation
F1	9.50e-75	4.56e-74	**1.22e-92**	**6.17e-92**
F2	1.03e-48	5.66e-48	**1.22e-67**	**6.70e-67**
F3	0.004596	0.006107	**0.002873**	**0.003752**
F4	1.89e-15	1.03e-14	**0**	**0**
F5	5.03e-15	**2.30e-15**	**4.08e-15**	2.69e-15
F6	0.014974	0.082019	**0.005092**	**0.027890**
F7	0.397901	4.87e-05	**0.397890**	**5.00e-06**
F8	-3.824472	0.141283	**-3.856691**	**0.012439**

Table 3 shows that in F1, F2, F3, F4, F6, F7 and F8, both WOA and PWOA have reached the target optimal value, but the average value and standard deviation of the optimal fitness obtained by PWOA are both superior to WOA. This demonstrates that PWOA has greater convergence accuracy and algorithm stability compared with WOA. In F5, however, the standard deviation of the optimal fitness of PWOA is higher than that of WOA, indicating that the stability of PWOA is slightly worse. On the other hand, both WOA and PWOA eventually converge to the optimal value, and the average value of the optimal fitness of PWOA is better, which also reflects the advantages of PWOA.

It can be seen from Fig 5 that in F3, F5, F7 and F8, when the difference between WOA and PWOA in the final convergence accuracy is not large, PWOA finds the optimal solution in the case of fewer iterations, indicating that PWOA has an excellent convergence speed compared to the original WOA. In F1, F2, F4 and F6, when the number of iterations is the same, the convergence accuracy of PWOA is higher than that of the original WOA.

Because the improved strategy of PWOA is to pair agents in the population in advance, the paired agents can share the information found in each iteration and help each other to move towards a better agent position. This strategy is equivalent to one agent exploring the search space twice in one iteration, which is more efficient. With the same parameters, PWOA is naturally superior to the original WOA. In general, PWOA improves the exploitation capability of meta-heuristic algorithms. Compared with the original WOA, the convergence speed and the convergence accuracy of PWOA have been improved to a certain extent. This further demonstrates the superiority and efficiency of PWOA, and PWOA as an efficient optimizer for experimental functions.

### Experimental results and analysis of PWOQLA

**Fig 6:**
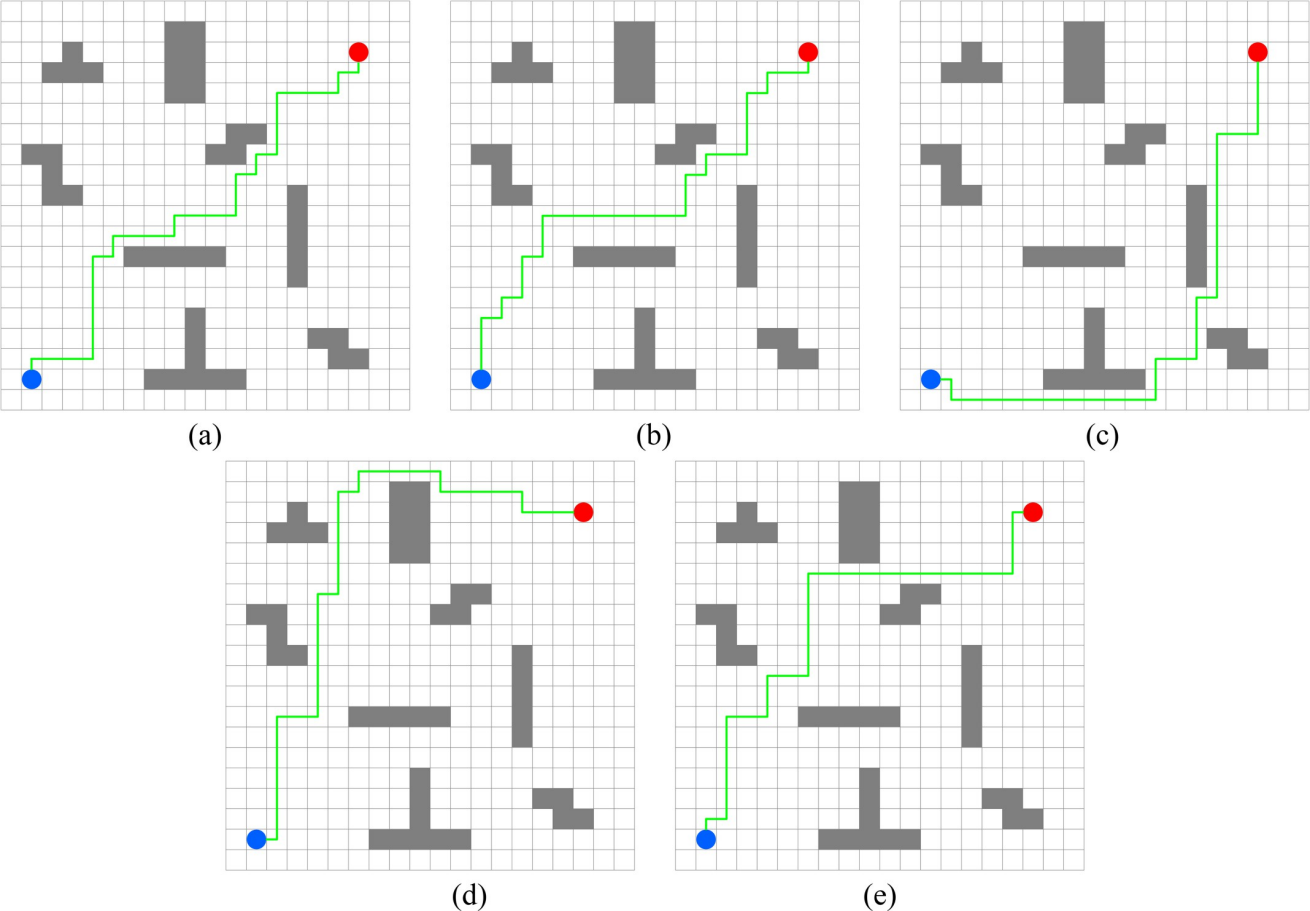
Best paths obtained by 5 algorithms in 6 experiments.

**Fig 7:**
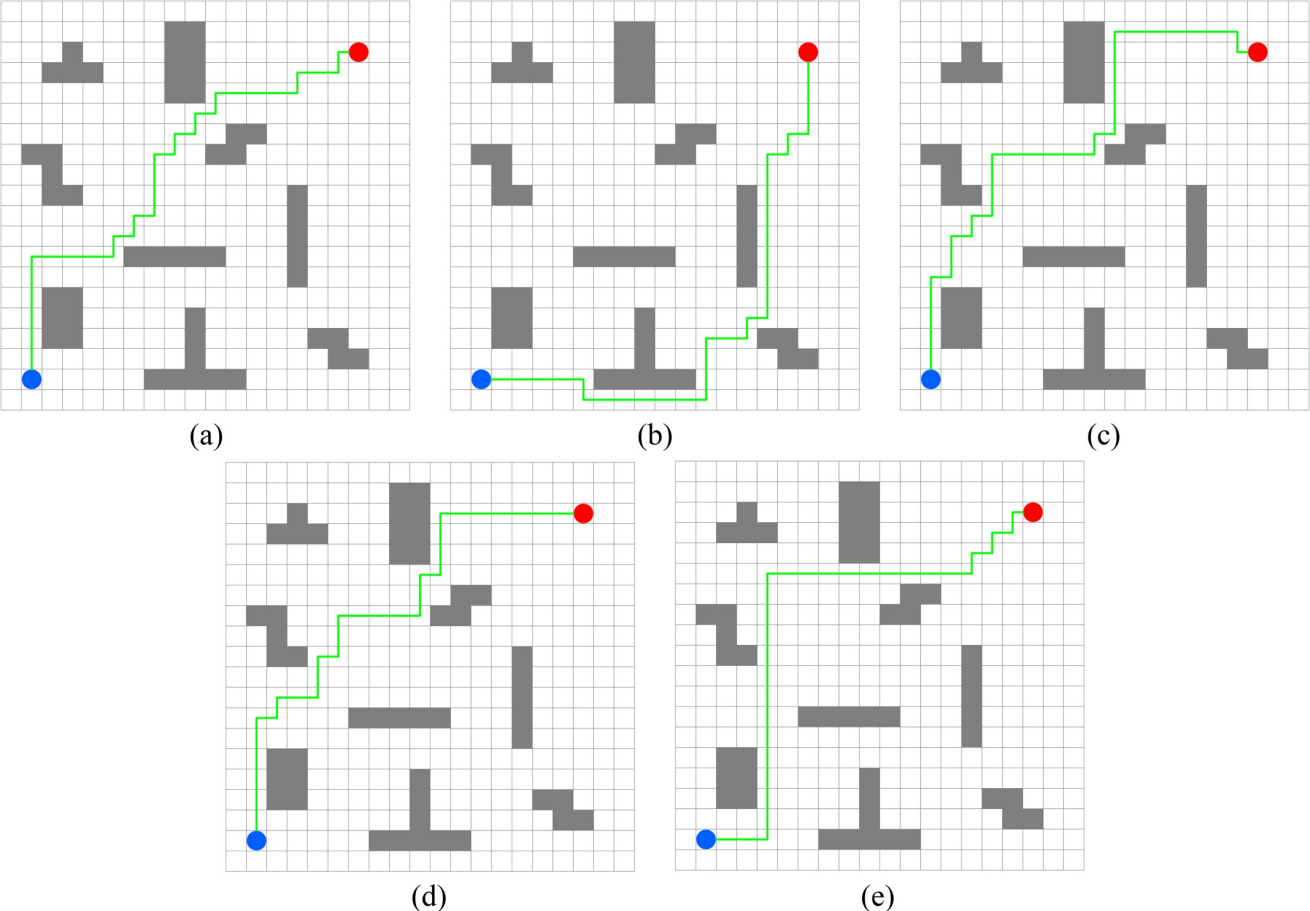
Best paths obtained by 5 algorithms in 6 experiments.

**Fig 8:**
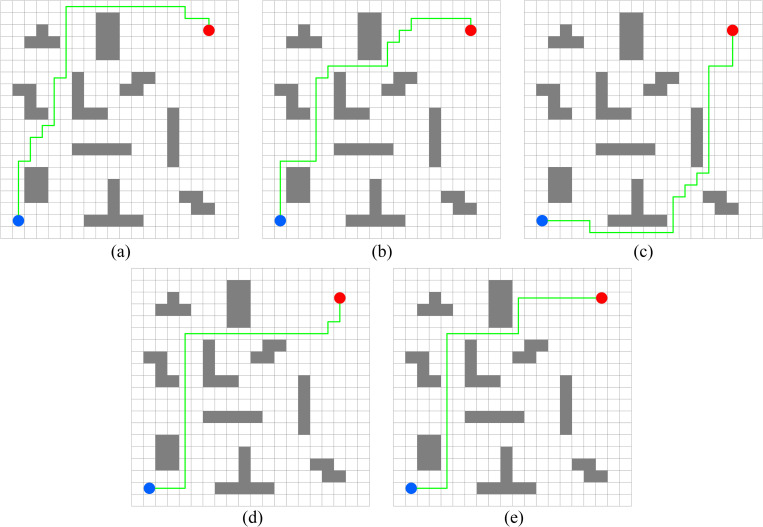
Best paths obtained by 5 algorithms in 6 experiments.

**Fig 9:**
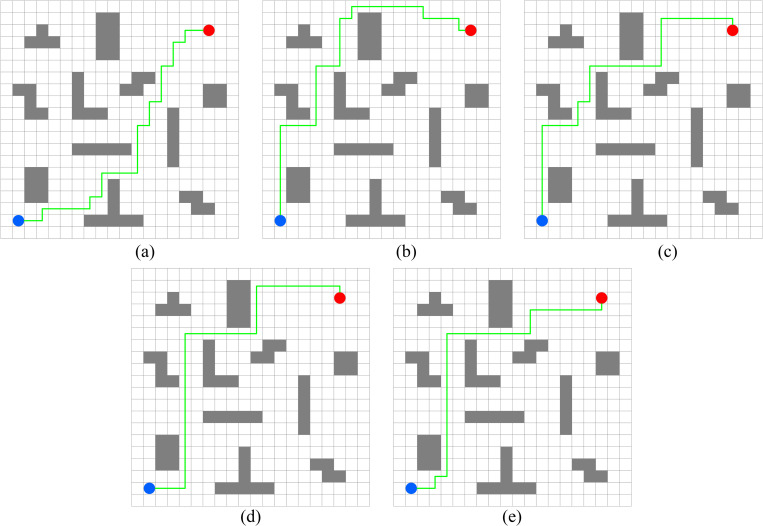
Best paths obtained by 5 algorithms in 6 experiments.

**Fig 10:**
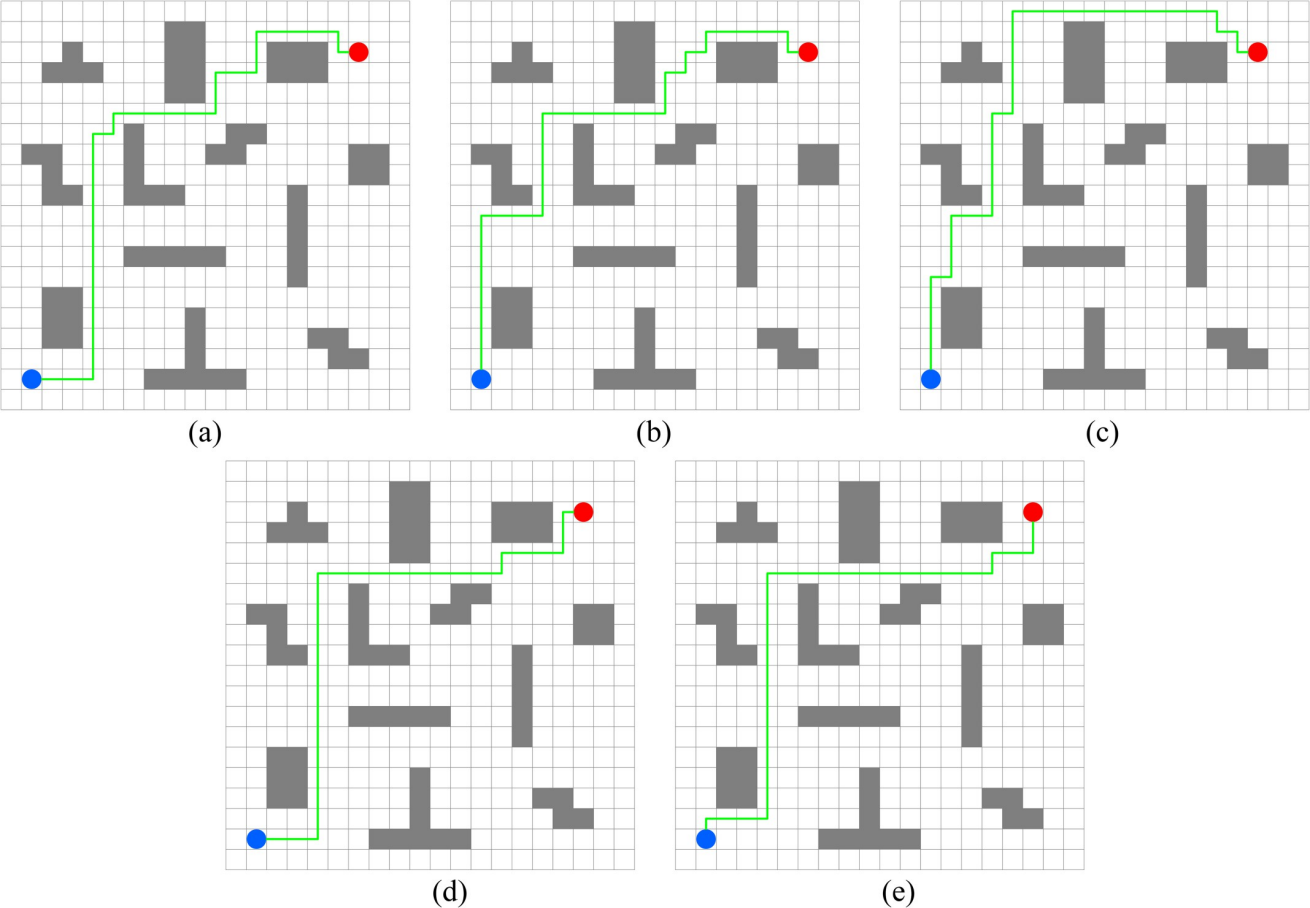
Best paths obtained by 5 algorithms in 6 experiments.

**Fig 11:**
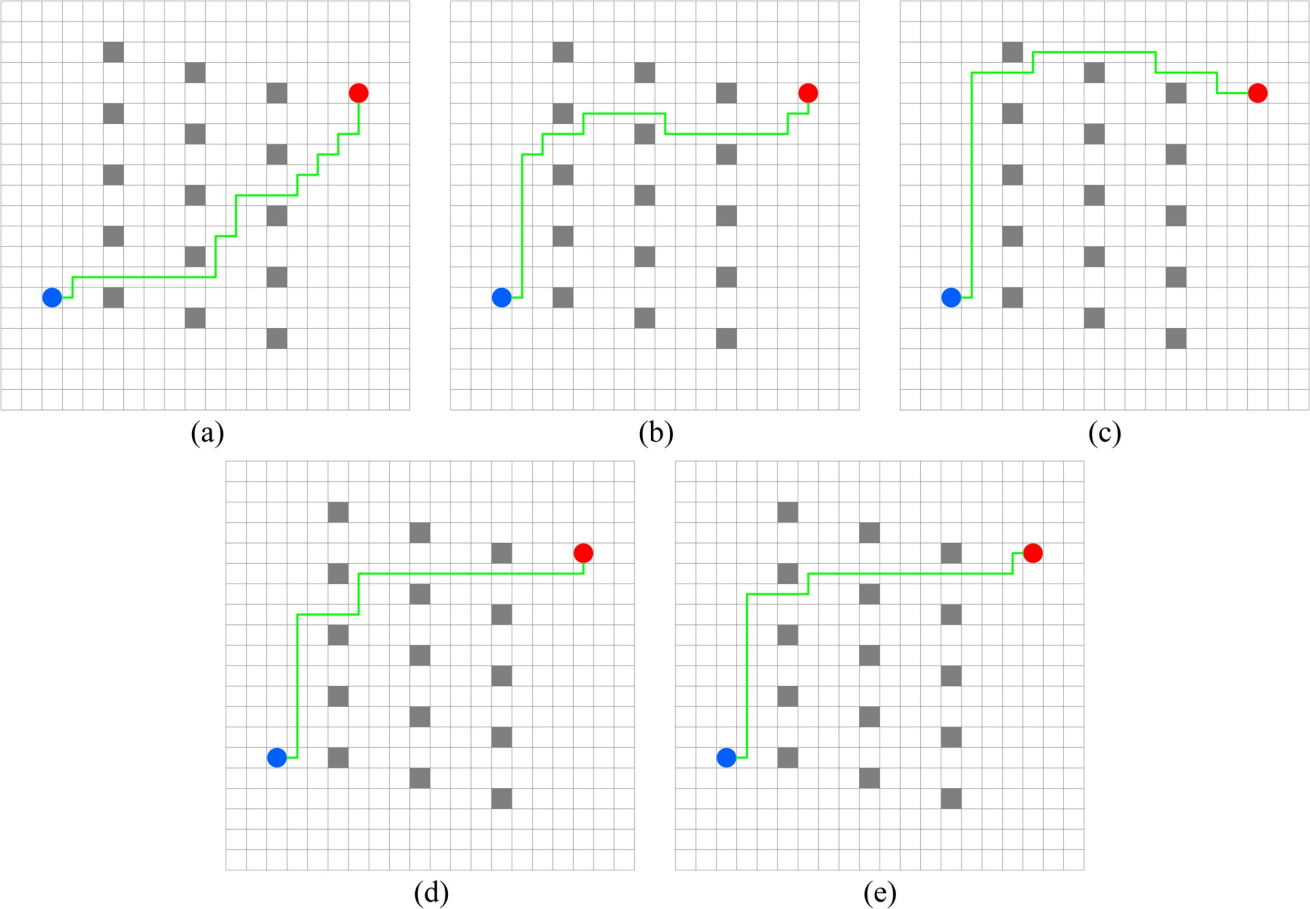
Best paths obtained by 5 algorithms in 6 experiments.

**Fig 12:**
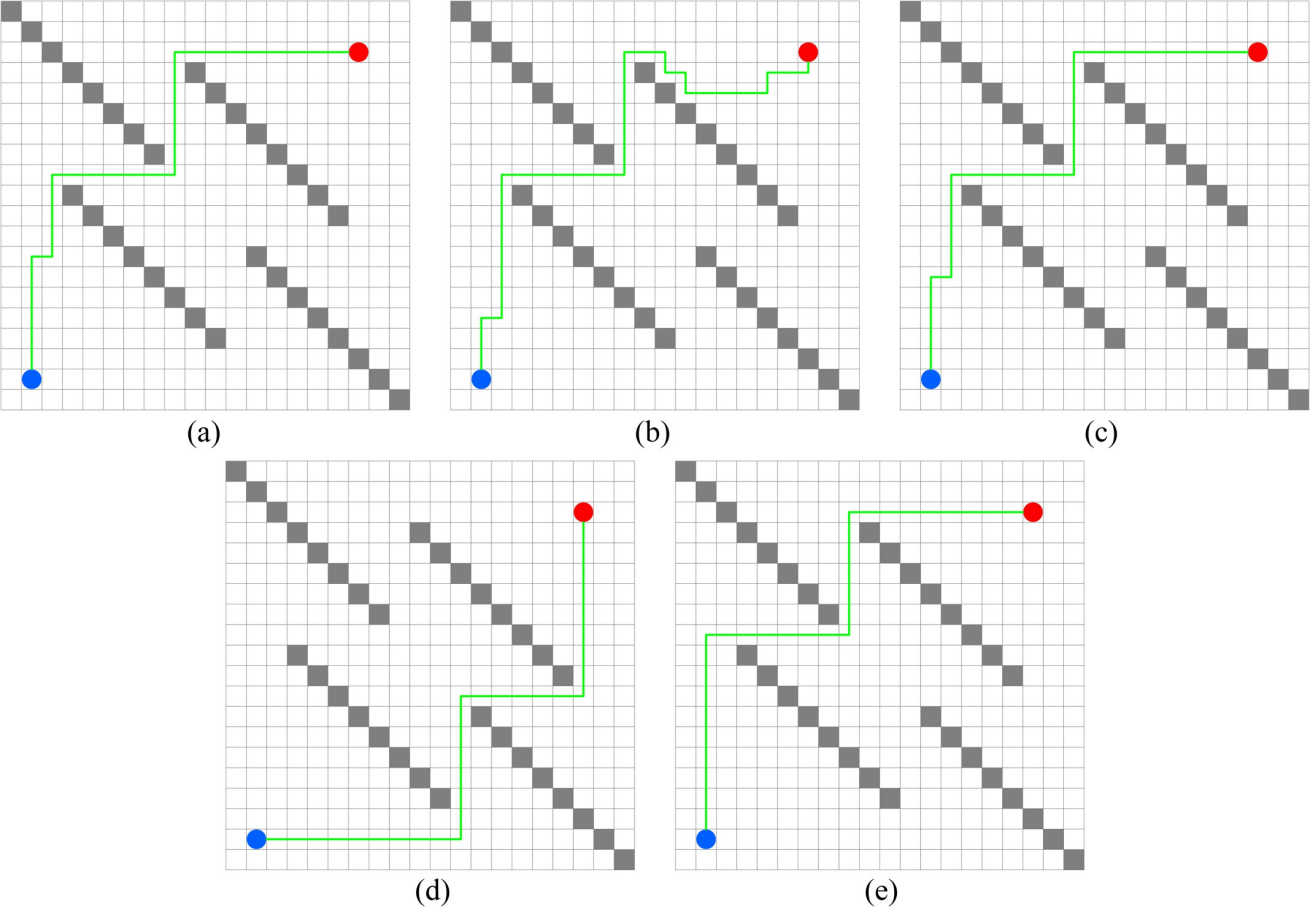
Best paths obtained by 5 algorithms in 6 experiments.

**Fig 13:**
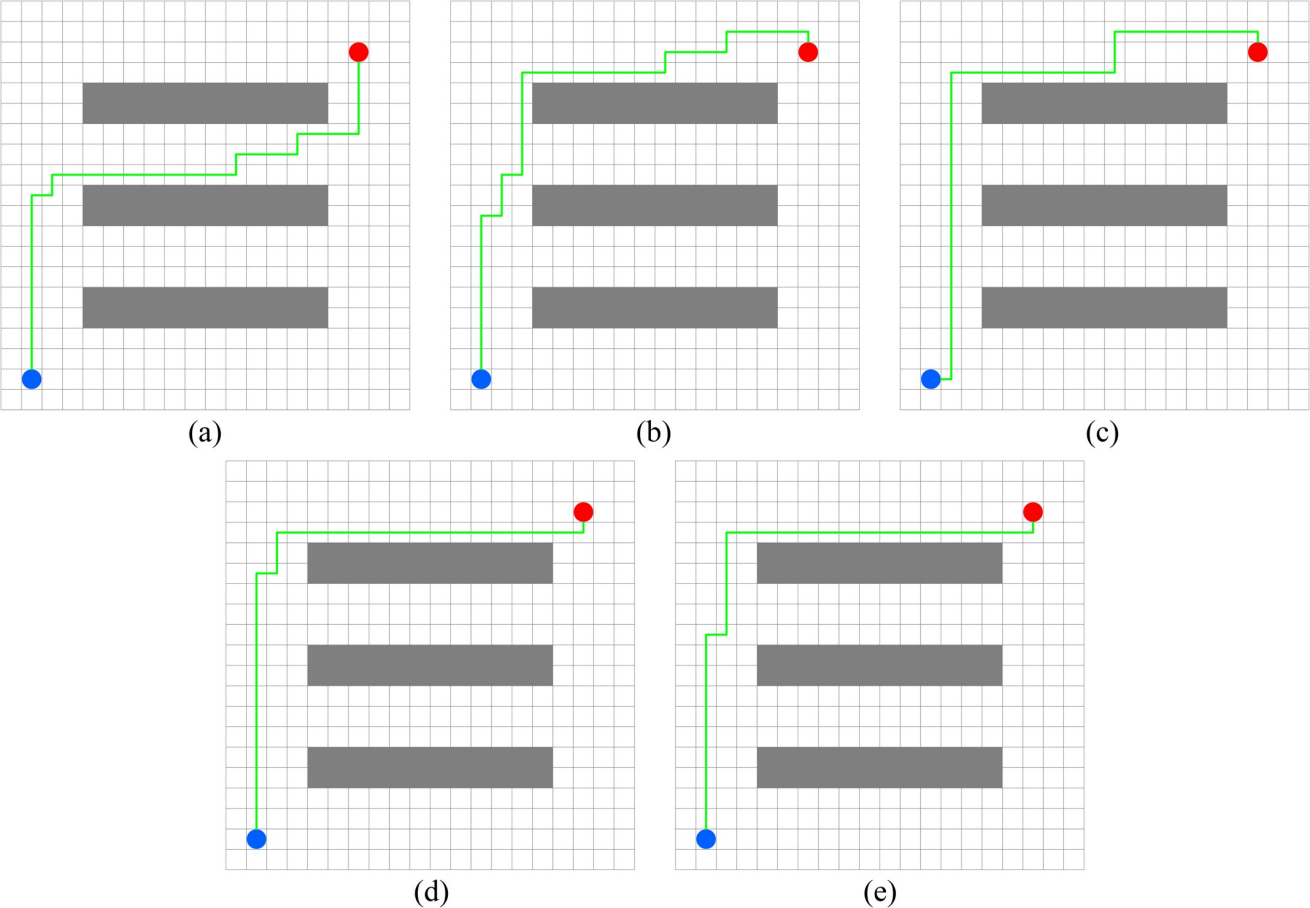
Best paths obtained by 5 algorithms in 6 experiments.

**Fig 14:**
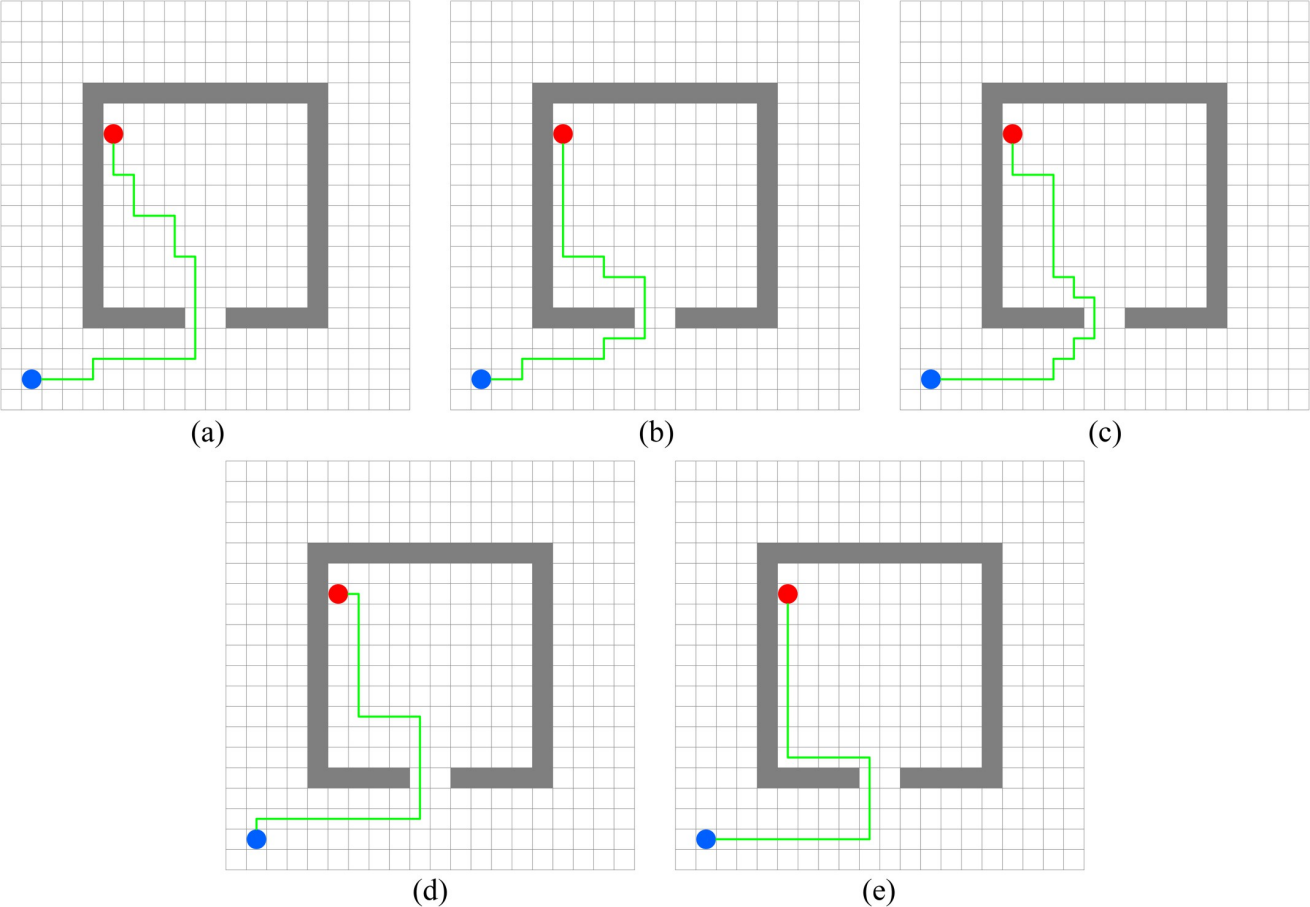
Best paths obtained by 5 algorithms in 6 experiments.

**Fig 15:**
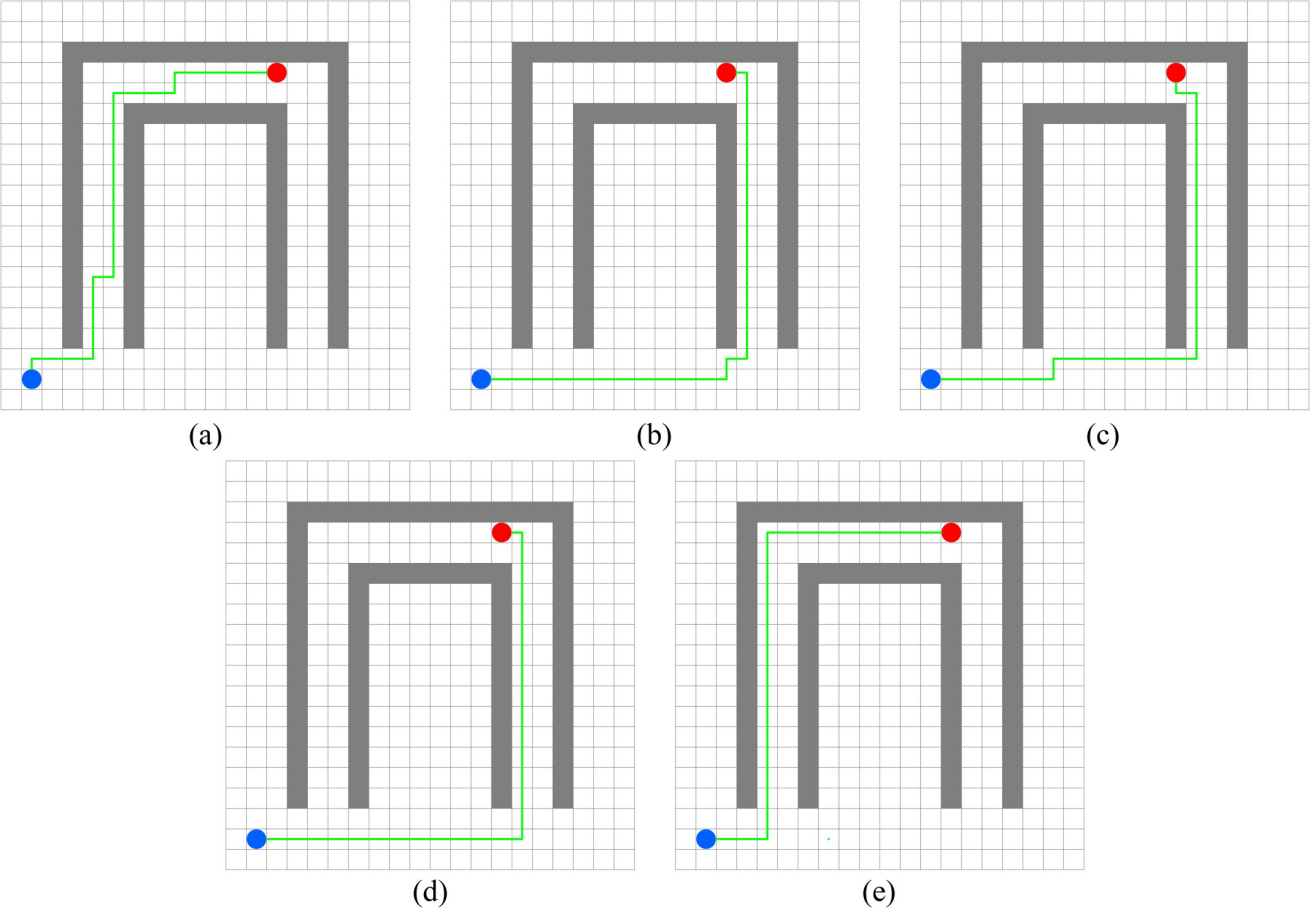
Best paths obtained by 5 algorithms in 6 experiments.

In this experiment, the performance of PWOQLA is compared with that of the original Q-learning, IDQ, A*algorithm, and WOQLA. We compare the averages and standard deviations of the running time, the number of path steps, and the number of rotation angles during 30 simulations, and analyze features, advantages, and disadvantages. The running time simply and directly reflects the efficiency of the algorithm: the shorter the time, the smaller the complexity of the algorithm, and the higher the efficiency. Because it is a square grid with the same distance between each node, the comparison of the number of path steps is equivalent to a comparison of the path length. Finding the shortest path is one of the ultimate goals of the path planning algorithm. Finally, the number of rotation angles is calculated to obtain the steering situation of the mobile robot under actual conditions. If the number of rotation angles is small, it means that the path is smoother, and that the mobile robot traveling along the path does not need frequent changes of direction, which means that the path planning algorithm is naturally more efficient. Figs 6–15 show the best paths obtained respectively by the A* algorithm, Q-learning, IDQ, WOQLA and PWOQLA in 6 experiments composed of different types of grid maps. In these figures, the blue dot represents the starting point, the red dot represents the destination, the grey squares represent the obstacles, the white squares represent free space, and the green line represents the final path.

#### Experiment 1: Irregular obstacles

The aim of Experiment 1 is to study the path planning ability of PWOQLA when navigating irregular obstacles. The maps of the experiment are composed of 8–12 different irregular obstacles randomly distributed. The illustrations (a), (b), (c), (d) and (e) in Figs 6–10 show the best paths for path planning using the A* algorithm, Q-learning, IDQ, WOQLA and PWOQLA on the irregular obstacles map. Table 4 shows a comparison of the time to calculate the target position in the maps. Table 5 compares the number of path steps, and Table 6 compares the number of rotation angles.

**Table 4:** Comparison of experimental results in computational time for 8–12 irregular obstacles.

Algorithm	8 irregular obstacles		9 irregular obstacles		10 irregular obstacles		11 irregular obstacles		12 irregular obstacles	
	Avg	SD	Avg	SD	Avg	SD	Avg	SD	Avg	SD
A*	0.23	0.02	0.20	0.08	0.19	0.03	0.19	0.04	0.18	0.07
Q-learning	1.02	0.21	1.00	0.14	0.99	0.17	0.99	0.18	0.96	0.17
IDQ	1.12	0.38	1.15	0.41	1.18	0.22	1.22	0.46	1.25	0.43
WOQLA	0.92	0.15	0.99	0.08	1.03	0.11	1.05	0.09	1.06	0.13
PWOQLA	0.89	0.04	0.91	0.03	0.91	0.04	0.92	0.10	0.95	0.05

**Table 5:** Comparison of experimental results in path steps for 8–12 irregular obstacles.

Algorithm	8 irregular obstacles		9 irregular obstacles		10 irregular obstacles		11 irregular obstacles		12 irregular obstacles	
	Avg	SD	Avg	SD	Avg	SD	Avg	SD	Avg	SD
A*	31.07	0.37	31.00	0.00	31.00	0.00	31.07	0.37	31.00	0.00
Q-learning	31.07	0.37	31.13	0.51	31.07	0.37	31.20	0.61	31.13	0.51
IDQ	31.00	0.00	31.00	0.00	31.00	0.00	31.07	0.37	31.07	0.37
WOQLA	31.00	0.00	31.07	0.37	31.00	0.00	31.00	0.00	31.00	0.00
PWOQLA	31.00	0.00	31.00	0.00	31.00	0.00	31.00	0.00	31.00	0.00

**Table 6:** Comparison of experimental results in number of rotation angles for 8–12 irregular obstacles.

Algorithm	8 irregular obstacles		9 irregular obstacles		10 irregular obstacles		11 irregular obstacles		12 irregular obstacles	
	Avg	SD	Avg	SD	Avg	SD	Avg	SD	Avg	SD
A*	1281.00	264.56	1308.00	291.84	1287.00	272.29	1317.00	301.13	1290.00	281.79
Q-learning	1218.00	235.68	1203.00	247.31	1239.00	232.40	1185.00	281.73	1284.00	253.25
IDQ	1263.00	221.47	1196.00	281.19	1152.00	276.42	1251.00	248.67	1176.00	259.82
WOQLA	1161.00	234.31	1203.00	264.94	1134.00	229.80	1197.00	236.36	1128.00	241.71
PWOQLA	1131.11	221.17	1152.00	245.86	1116.00	218.77	1098.00	211.63	1104.00	223.49

It can be seen from Table 4 that although the calculation time of the A* algorithm is shortest, the cost function *f(n)* of the A* algorithm only considers the target position and does not consider the obstacles in the map. This leads to the A* algorithm having the largest average and the largest standard deviation of the number of rotation angles, as can be seen from Table 6, which shows that the path smoothness of the A* algorithm is the worst.

Table 4 also shows that, for the original Q-learning, when the number of obstacles increases, the calculation time gradually decreases. This is because the number of obstacles increases while the exploration space decreases, which saves time. In contrast, for IDQ, the calculation time gradually increases. This is because a greater number of local optimal solutions may be generated with the increase in the number of obstacles, which wastes time in this case. The calculation time for WOQLA and PWOQLA also increases with additional obstacles. When there are more obstacles in the map, the calculation time increases because early initialization may entrap Q-learning in a local optimal solution. When there are fewer obstacles in the map, the possibility of being trapped in a local optimal solution is reduced.

In general, PWOQLA achieves the shortest operation time among the algorithms except for the A* algorithm, followed by WOQLA and the original Q-learning, while IDQ has the longest operation time. According to Table 5, the average step values of the original Q-learning and IDQ are slightly higher than the other algorithms, while the average step value of PWOQLA is the shortest. These results show that in a disordered and irregular obstacle map, PWOQLA has the greatest path planning efficiency because it first optimizes the initialization of the Q-table, which simplifies the subsequent search strategy and accelerates the convergence speed.

#### Experiment 2: Lattice obstacles

Experiment 2 sets out to verify the path planning ability of PWOQLA on a regular lattice map. Fig 11 shows a diagram of the optimal path result. Table 7 shows a comparison of the time to find the target position, the path step length, and the number of rotation angles for each algorithm in the experiment. Lattice obstacles test mainly the smoothness of the planning path. It can be seen from Fig 11 and Table 7 that the path planned by PWOQLA is the smoothest, with the shortest computational time and step value. Compared with the A* algorithm, the average number of rotation angles for PWOQLA is an improvement of 31.6%. Compared with the original Q-learning, the average number of rotation angles for PWOQLA is an improvement of 31.0%. The standard deviation in the number of rotation angles is also the smallest for PWOQLA, indicating that the path of PWOQLA is more stable and always finds the target with fewer rotations.

**Table 7:** Comparison of experimental results in computational time, path steps and number of rotation angles for lattice obstacles.

Algorithm	Computational Time		Path Steps		Number of Rotation Angles	
	Avg	SD	Avg	SD	Avg	SD
A*	0.22	0.05	24.00	0.00	1101.00	275.65
Q-learning	1.03	0.09	24.13	0.51	1020.00	214.46
IDQ	1.00	0.08	24.07	0.37	951.00	232.11
WOQLA	0.96	0.03	24.00	0.00	825.00	210.27
PWOQLA	0.86	0.06	24.00	0.00	753.00	198.75

#### Experiment 3: Strip obstacles

Experiment 3 studies the path planning of each algorithm when the initial position and target position are separated by multiple long-strip obstacles and the target cannot be reached directly. Fig 12 shows a diagram of the optimal path result. Table 8 shows the experimental results for each algorithm. Because the terrain is too complex, and the path to the destination needs to pass no less than 3 obstacles, the original Q-learning has the longest calculation time. The calculation time for PWOQLA is still the shortest. The average number of path rotation angles for IDQ, WOQLA and PWOQLA are all less than the original Q-learning, indicating that these algorithms have improved the smoothness of the optimized path. Their standard deviations in the number of rotation angles has also been reduced, indicating that the path is more stable.

**Table 8:** Comparison of experimental results in computational time, path steps and number of rotation angles for strip obstacles.

Algorithm	Computational Time		Path Steps		Number of Rotation Angles	
	Avg	SD	Avg	SD	Avg	SD
A*	0.20	0.04	31.00	0.00	539.00	93.68
Q-learning	1.02	0.06	31.07	0.37	528.00	86.94
IDQ	0.92	0.05	31.00	0.00	426.00	83.51
WOQLA	0.91	0.06	31.07	0.37	435.00	81.15
PWOQLA	0.89	0.10	31.00	0.00	393.00	78.88

#### Experiment 4: Horizontal obstacles

Experiment 4 sets out to test the ability of these algorithms to pass through straight and narrow paths. From Table 9, it can be seen that the A* algorithm, Q-learning and IDQ cannot find the best path via the shortest route in each simulation, whereas WOQLA and PWOQLA perform well in this respect. Although WOQLA and PWOQLA have no advantage in computing time, the number of rotation angles has been significantly reduced. Compared with the original Q-learning, the number of rotation angles for WOQLA is reduced by 25.6% and the number for PWOQLA is reduced by 32.4%. Additionally, Fig 13 shows that WOQLA and PWOQLA take smoother paths and fewer turns to reach the destination. If the algorithm is applied to the path planning of mobile robots, these algorithms will reduce the time for the mobile robot to rotate and change direction, and save resources.

**Table 9:** Comparison of experimental results in computational time, path steps and number of rotation angles for horizontal obstacles.

Algorithm	Computational Time		Path Steps		Number of Rotation Angles	
	Avg	SD	Avg	SD	Avg	SD
A*	0.30	0.12	31.00	0.00	810.00	242.98
Q-learning	0.90	0.09	31.13	0.51	795.00	203.98
IDQ	0.91	0.06	31.07	0.37	798.00	190.55
WOQLA	0.93	0.05	31.00	0.00	591.00	109.05
PWOQLA	0.96	0.09	31.00	0.00	537.00	99.18

#### Experiment 5: Room type obstacles

Experiment 5 is a simulation of finding the target position in a room and testing the path planning ability of the algorithms when there are room-type obstacles. It can be seen from Fig 14 that the path found by PWOQLA is the best visible to the naked eye. Table 10 indicates that, except for the A* algorithm, PWOQLA has the shortest average operation time and is the most suitable for this scenario, while each algorithm can find the path with stability and the least number of steps every time.

**Table 10:** Comparison of experimental results in computational time, path steps and number of rotation angles for room type obstacles.

Algorithm	Computational Time		Path Steps		Number of Rotation Angles	
	Avg	SD	Avg	SD	Avg	SD
A*	0.20	0.05	23.00	0.00	870.00	195.46
Q-learning	1.17	0.45	23.00	0.00	822.00	173.21
IDQ	0.92	0.06	23.00	0.00	801.00	177.98
WOQLA	0.93	0.08	23.00	0.00	789.00	211.28
PWOQLA	0.90	0.08	23.00	0.00	816.00	199.90

#### Experiment 6: Concave obstacles

The aim of Experiment 6 is to simulate finding the target position in a narrow concave tunnel. Combining Fig 15 and Table 11, it can be seen that the average number of rotation angles of the original Q-learning, IDQ, WOQLA and PWOQLA are almost the same, while the standard deviations are not much different, indicating that these algorithms are suited to this kind of scene. However, compared to other scenarios, the best path with the least number of steps cannot be found every time. In the Experiment 6 scenario, the A* algorithm and the original Q-learning will take detours to find the target position during some simulations, whereas IDQ, WOQLA and PWOQLA are more stable, being able to find the path with the least number of steps more often.

**Table 11:** Comparison of experimental results in computational time, path steps and number of rotation angles for concave obstacles.

Algorithm	Computational Time		Path Steps		Number of Rotation Angles	
	Avg	SD	Avg	SD	Avg	SD
A*	0.18	0.09	26.40	0.81	674.00	180.25
Q-learning	0.90	0.10	26.27	0.70	672.00	145.53
IDQ	0.95	0.09	26.07	0.37	651.00	173.93
WOQLA	0.88	0.09	26.07	0.37	645.00	140.60
PWOQLA	0.85	0.05	26.07	0.37	636.00	112.65

#### Wilcoxon rank-sum test

The Wilcoxon rank-sum test is a nonparametric hypothesis test, which is used to infer whether there is a difference between the distribution positions of two populations. It reflects the correlation of the experimental results of each algorithm in 30 independent runs. In this test, a p-value with a 95% significance level was computed, which means that when the test value is less than 0.05, it indicates that there is a significant difference between the experimental data of different algorithms. And the corresponding results for computational time, path steps and number of rotation angles are reported in Table 12.

**Table 12:** Wilcoxon rank-sum test.

Maps	Indexes	PWOQLA vs. A*	PWOQLA vs. Q-learning	PWOQLA vs. IDQ	PWOQLA vs. WOQLA
8 irregular obstacles	Computational Time	4.98e-10	3.63e-04	6.68e-06	5.48e-04
	Path Steps	3.34e-01	3.34e-01	NaN	NaN
	Number of Rotation Angles	3.89e-02	1.45e-01	5.22e-02	1.33e-01
9 irregular obstacles	Computational Time	2.99e-11	9.12e-03	7.23e-04	4.10e-02
	Path Steps	NaN	1.61e-01	NaN	3.34e-01
	Number of Rotation Angles	5.62e-03	5.62e-02	5.56e-01	7.65e-02
10 irregular obstacles	Computational Time	2.87e-11	4.38e-03	9.76e-05	8.87e-04
	Path Steps	NaN	3.34e-01	NaN	NaN
	Number of Rotation Angles	4.66e-02	5.92e-02	7.66e-02	1.96e-01
11 irregular obstacles	Computational Time	2.91e-11	1.07e-03	4.35e-04	9.12e-04
	Path Steps	3.34e-01	8.14e-02	3.34e-01	NaN
	Number of Rotation Angles	6.32e-02	1.17e-01	6.12e-02	1.01e-01
12 irregular obstacles	Computational Time	2.86e-11	3.44e-04	6.68e-05	1.19e-05
	Path Steps	NaN	1.61e-01	3.34e-01	NaN
	Number of Rotation Angles	3.26e-03	1.24e-02	3.81e-02	7.69e-02
lattice obstacles	Computational Time	2.98e-11	3.33e-08	1.78e-07	4.78e-04
	Path Steps	NaN	1.61e-01	3.34e-01	NaN
	Number of Rotation Angles	1.94e-04	1.03e-01	1.04e-02	2.23e-02
strip obstacles	Computational Time	2.91e-11	3.20e-03	3.43e-02	3.00e-02
	Path Steps	NaN	3.34e-01	NaN	3.34e-01
	Number of Rotation Angles	6.27e-01	8.75e-02	7.59e-02	5.49e-01
horizontal obstacles	Computational Time	2.97e-11	9.76e-05	3.20e-03	6.09e-04
	Path Steps	NaN	1.61e-01	3.34e-01	NaN
	Number of Rotation Angles	2.72e-02	9.25e-03	3.58e-04	2.12e-02
type obstacles	Computational Time	2.94e-11	5.74e-04	1.83e-03	4.41e-03
	Path Steps	NaN	NaN	NaN	NaN
	Number of Rotation Angles	3.02e-02	5.06e-02	1.48e-02	6.80e-01
concave obstacles	Computational Time	2.93e-11	7.36e-03	4.75e-02	6.90e-04
	Path Steps	4.76e-02	1.69e-01	1.00e-00	1.00e-00
	Number of Rotation Angles	8.24e-04	2.40e-02	1.04e-03	6.54e-02

According to Table 12, it can be seen that under different experimental map environments, compared with other path planning algorithms, data distribution on computational time is significantly different in PWOQLA, which indicates that PWOQLA has significantly improved the path planning time, proving its superiority. There is no significant difference between some data of path steps and number of rotation angles. First, due to the limitation of data types, and second, because other algorithms are excellent enough in the performance of these two test indicators, so the results in Table 12 are obtained.

## Discussion

In general, except for the A* algorithm, PWOQLA has the best performance in these experiments, which is shown in the shortest calculation time and the smoothest path. The reason why PWOQLA has the best performance is that it uses PWOA to improve the initialization process of the original algorithm, so that the initialized Q-table contains some previous learning experience. At the same time, PWOQLA algorithm uses SES strategy ε dynamic curve to helps reduce the number of useless explorations. The calculation time for the A* algorithm is shorter because the A* algorithm is a direct search algorithm, whereas the other four algorithms based on the original Q-learning are reinforcement learning algorithms. Reinforcement learning algorithms require more learning time than direct search algorithms. Therefore, although the calculation time of the A* algorithm is shorter, the path that it generates has a larger number of steps and a larger number of rotation angles. Considering the actual requirements for mobile robot path planning, PWOQLA is obviously better. In Experiment 4, the calculation time of PWOQLA is longest in five simulations. The reason why PWOQLA does not perform well in Experiment 4 is that the exploitation ability of PWOQLA has not been improved. When the obstacle area is relatively large and concentrated, the disadvantages of PWOQLA are more obvious. PWOQLA focuses more on improving the exploration strategy, mainly improving the calculation time of the algorithm. In Experiment 3, however, the path planned by PWOQLA is the smoothest, and both the average and the standard deviation of the rotation angles are the smallest. This shows that the pre-treatment during PWOQLA initialization compensates for the exploitation ability to a certain extent, enhancing an understanding of the map, and helping PWOQLA to find the optimal path.

Comparing PWOQLA performances in all of these experiments, Experiment 6 is especially notable. In navigating concave obstacles, PWOQLA takes the shortest time in five simulations to find the target, with an average of only 0.85s and a standard deviation of 0.05s, which is the shortest too. The results of Experiment 6 show that PWOQLA performs best in computational time, indicating that it is the most suitable for the path planning of mobile robots in concave obstacle maps similar to this experiment, such as maps with many curves or narrow tunnels. In Experiment 4 on the other hand, PWOQLA takes the longest time compared with other PWOQLA performances, with an average computational time of 0.96s. This shows that PWOQLA is not the best performer when obstacles are regular and repeated. Thus, the exploitation capability of PWOQLA should be further improved.

Although the exploitation capability of PWOQLA is insufficient, the experimental results show that PWOQLA still meets the requirements of speeding up path planning time and finding the best path with fewer rotation angles. Moreover, PWOQLA overcomes the disadvantage of slower convergence in the original Q-learning.

## Conclusions

The convergence speed Q-learning is slow because is too simple when initializing the Q-table and wastes too much time in the exploration process. To solve these problems, we propose PWOQLA. Firstly, the WOQLA proposal solves the problem of slow convergence speed caused by the simple initialization of the Q-table. Through this innovation, in which the original WOA is used to initialize the values of the Q-table, a Q-table containing previous experience is obtained before the exploration process. Thus, the convergence speed of *ε*-greedy Q-learning is accelerated. Secondly, the PWOA proposal speeds up the speed of the whale population approaching the local optimal solution, solving the shortcoming of slow convergence in the original WOA. Thus, the efficiency of Q-learning initialization in WOQLA can be improved by replacing WOA with PWOA. Thirdly, the SES proposal, which utilizes the position relationship between the current agent and the target, reduces the useless exploration of *ε*-greedy Q-learning and further improves the convergence speed. Fourthly, the proposal of a dynamically changing nonlinear function for *ε* overcomes the shortcoming that exploration and exploitation cannot be switched flexibly in the original *ε*-greedy Q-learning. Experimental results show that PWOQLA has greater accuracy and faster convergence speed compared with algorithms with similar functions.

Although PWOQLA balances exploration and exploitation capability in a static environment, the exploitation capability of PWOQLA is insufficient in a dynamic environment. Thus, PWOQLA could be combined with other algorithms that have strong exploitation capabilities when applied to path planning in dynamic or extreme environments. The method of determining the *ε* dynamic curve parameters in PWOQLA can also be further improved. In future work, we will apply PWOQLA to mobile robot path planning in dynamic or extreme environments and test its performance.
